# A dataset on radical and extremist attitudes in Europe

**DOI:** 10.1038/s41597-026-08455-x

**Published:** 2026-10-17

**Authors:** Sebastian Jungkunz, Nina Fadarkhan Osenbrügge, Marc Helbling

**Affiliations:** 1 Martin Luther University Halle-Wittenberg, Halle (Saale), Germany; 2 University of Bamberg, Bamberg, Germany; 3 University of Mannheim, Mannheim, Germany

## Abstract

This dataset introduces the first cross-nationally validated measurement scales for left-wing and right-wing radicalism, and general political extremism, along with an implicit association test (IAT) for implicit political extremism in the Western world. It includes data from more than 18,000 participants collected through ten surveys conducted between 2022 and 2024 in France, Germany, Great Britain, Greece, Hungary, the Netherlands, and the United States. The dataset facilitates comparative analyses of the origins and consequences of radical and extremist attitudes across different political systems. Moreover, by linking attitudinal measures with behavioral indicators such as party support, it provides a foundation for examining how latent forms of extremism are translated into political action. Beyond extremism research, the dataset contributes to the broader study of democratic resilience, political polarization, and ideological belief structures.

## Background & Summary

In recent years, political radicalism and extremism have moved from the fringes to the center of public debate across the Western world^1–4^. Parties and movements that reject or challenge liberal-democratic norms have gained visibility and influence, reshaping electoral competition and public discourse. While populist parties have been widely studied, their overlap with genuinely extremist organizations and supporters – those who oppose constitutional democracy itself – remains poorly understood. Addressing this gap requires not only conceptual clarity but also valid and comparable empirical tools to measure extremist attitudes across societies.

Political extremism refers to attitudes that reject the fundamental principles of the constitutional democratic state and seek to replace them with an alternative political order^5,6^. At its core, extremism is characterized by anti-democratic orientations that reject pluralism, political opposition, and democratic contestation as illegitimate. Extremist worldviews are typically built upon a claim to absolute truth, dogmatism, dualistic friend–foe distinctions, holistic and deterministic conceptions of society and history, identitarian constructions of the political community, and a fundamental rejection of the existing democratic order^5^. These characteristics are not confined to a particular ideological position but can emerge across the political spectrum, including left-wing, right-wing, and religiously motivated forms of extremism, all of which share a rejection of constitutional democracy and its core institutions.

Religious fundamentalism overlaps conceptually with political extremism in its absolutist and anti-pluralistic orientation but differs in its primary point of reference. Rather than centering on political ideology, religious fundamentalism is grounded in the belief that a single body of religious teachings constitutes the infallible truth about humanity and the divine, that this truth is threatened by forces of evil, and that these threats must be actively resisted^7,8^. Similar to political extremism, religious fundamentalism rejects individual autonomy and the fundamental equality of all human beings, thereby conflicting with core liberal-democratic principles.

Political radicalism, in contrast, accepts the procedural rules of democracy while opposing key normative foundations of liberal democracy^9^. Radicals advocate far-reaching political and societal transformation without necessarily rejecting democratic institutions themselves. Right-wing radicalism emphasizes nationalism, exclusionary conceptions of political community, and hierarchical understandings of social groups, whereas left-wing radicalism extends egalitarian principles to the point of subordinating individual liberties to collective goals and is commonly expressed through support for socialism and anti-capitalist, anti-imperialist, and anti-fascist positions^6^. Although radicals do not inherently reject the constitutional democratic state, extremists frequently combine anti-democratic attitudes with radical ideological commitments. Distinguishing between radicalism and extremism is therefore essential for identifying when strong ideological convictions cross the threshold into opposition to democracy itself.

Finally, we conceptualize political extremism, radicalism, and religious fundamentalism primarily as attitudinal phenomena. Although these orientations may be associated with political violence or militant action, neither violence nor illegal behavior constitutes a defining feature of these concepts. Conflating extremist attitudes with violent behavior obscures important conceptual distinctions between extremism and terrorism^6,10^. Moreover, these attitudinal orientations may exist both as explicit and implicit evaluations. Whereas explicit attitudes reflect consciously accessible and self-reported beliefs, implicit attitudes represent automatic evaluations that operate outside conscious awareness and may influence political judgment and behavior without deliberate reflection^11^.

The distinction between explicit and implicit attitudes is particularly relevant for the study of political extremism. Because anti-democratic views are often socially undesirable in democratic societies, respondents may conceal or underreport extremist beliefs in conventional surveys^12^. Implicit attitudes therefore provide insight into latent ideological predispositions that remain inaccessible to self-report measures while nevertheless shaping political preferences and behavior^13–16^. Assessing both explicit and implicit attitudes offers a more comprehensive understanding of extremist orientations and their potential mobilization under changing political opportunities or shifting social norms^17^.

Existing research on political extremism has relied heavily on behavioral indicators such as vote choice for radical parties or self-placement on the left-right spectrum. These measures, however, fail to capture latent extremist dispositions. Voting behavior can reflect protest, dissatisfaction, or strategic considerations rather than anti-democratic convictions^18^. Likewise, left-right self-placement varies in meaning across individuals, groups, and countries; respondents who consider themselves “moderate” may nonetheless endorse extremist ideas^19,20^. Furthermore, the boundaries of party systems and ideological labels shift over time, making behavioral and positional measures unreliable for longitudinal or cross-national comparison^21–23^. Identifying extremism at the attitudinal level before it manifests in political action is therefore critical for understanding processes of radicalization and democratic resilience.

Despite decades of research, the field lacks standardized, validated, and cross-nationally comparable measures of political radicalism and extremism^24^. Previous instruments, developed mainly in single-country studies, often conflate extremism with related but distinct constructs such as authoritarianism, prejudice, or approval of political violence^25–27^. Others emphasize issue-specific positions, such as racism or anti-capitalism, without addressing their relationship to democratic principles^25,28^. As a result, it has remained unclear whether these measures truly identify anti-democratic worldviews or merely capture strong ideological preferences.

The present dataset fills this gap by providing a theoretically grounded and empirically validated tool to measure political extremism and radicalism across six European democracies. Building on previous work^17,29^, the data include newly developed and psychometrically tested scales for general extremism, right-wing radicalism, and left-wing radicalism, as well as an implicit association test (IAT) for implicit extremist attitudes along with an established measure for religious fundamentalism. The conceptual framework distinguishes between a core “extremist belief system”, defined by anti-democratic and anti-pluralist attitudes, and ideological expressions on the right and left that emphasize hierarchy between nations and ethnicities or superimpose equality in socio-political context to an illiberal extreme. This two-dimensional structure allows for the systematic differentiation of radicals, extremists, and non-extremists. By combining the radicalism and extremism scales, right- and left-wing extremist attitudes can also be studied.

The data was collected in six European countries (France, Germany, Great Britain, Greece, Hungary, and the Netherlands) and the United States, to ensure variation in political culture and exposure to radical movements. The surveys include extensive socio-demographic and attitudinal variables, enabling research on the psychological, social, and political correlates of extremism. The inclusion of both explicit and implicit measures allows scholars to study the discrepancy between self-reported and latent extremist attitudes, an issue of particular importance given social desirability pressures in surveys on sensitive topics.

This resource substantially advances research on political extremism in three ways. First, it offers the first cross-nationally validated scales for left- and right-wing radicalism and general extremism in the Western world that clearly distinguish between democratic dissent and anti-democratic rejection. Second, it enables comparative analyses of the antecedents and consequences of extremist attitudes across political systems [see^30^]. Third, by linking attitudinal data to behavioral indicators such as party support, it provides a foundation for investigating how latent extremism translates into political action. Beyond extremism research, the dataset contributes to the broader study of democratic resilience, polarization, and ideological belief systems, offering an empirically rigorous basis for monitoring threats to liberal democracy over time.

## Methods

### Participants

The dataset comprises 18,432 participants from 10 surveys across 7 countries, including three pretests (one in the United States and two in Germany). In 2022, the data for the first main study were collected in Germany, Great Britain, and the Netherlands, and in 2024, the second study was conducted in Germany, France, Greece, and Hungary. Except for the pre-test in the United States (Mturk), participants were recruited through online access panels of Bilendi & respondi using a double-opt-in registration process. Participation in the panel is voluntary, and participants provided their written informed consent before the study started. For the main studies, quota sampling was applied to reach representativeness in terms of age (18 to 69 years), gender, and educational level. Muslims were oversampled in Germany and the UK in Study 1 and in France and Germany in Study 2 of the main studies, as well as in one of the pretests in Germany. This oversampling allows the comparison of radicalism, extremism, and religious fundamentalism between Muslims and non-Muslims. All pretests and main studies were administered in the respective official language of the countries. For the multi-country studies, questionnaires were prepared in English and then translated by expert translators of the survey company into the respective national languages. Table 1 provides the respective fieldwork periods with survey length and summarizes age, gender, education, and religious affiliation across the samples.

**Table 1:** Sample characteristics by survey and country.

	Country	Fieldwork	Survey length	Age (Mean ± SD)	Female (%)	High Education (%)	Muslims (%)	N
Pretest 1	United States	2021‐05‐20 until 2021‐05‐21	14.2	40.44 ± 11.90	43.74	87.98	0.81	494
Pretest 2	Germany	2021‐09‐03 until 2021‐09‐11	26.2	42.44 ± 14.58	48.30	32.51	28.30	1797
Pretest 3	Germany	2022‐01‐11 until 2022‐01‐27	30.4	45.30 ± 14.33	50.26	22.24	1.91	1520
Study 1	Germany	2022‐06‐21 until 2022‐08‐15	34.3	45.46 ± 14.20	51.40	27.81	24.18	2117
Study 1	United Kingdom	2022‐06‐22 until 2022‐09‐13	35.3	46.27 ± 12.86	44.46	45.23	14.49	2039
Study 1	Netherlands	2022‐06‐22 until 2022‐09‐10	34.7	43.47 ± 15.75	56.95	21.55	4.25	2045
Study 2	Germany	2024‐02‐22 until 2024‐05‐29	23.5	41.94 ± 14.37	52.76	18.71	25.33	2100
Study 2	France	2024‐04‐02 until 2024‐07‐09	25.4	43.12 ± 13.57	55.14	49.57	25.00	2100
Study 2	Greece	2024‐04‐05 until 2024‐07‐04	22.8	43.83 ± 13.58	50.88	52.98	0.47	2150
Study 2	Hungary	2024‐04‐05 until 2024‐06‐27	24.4	44.31 ± 14.14	50.68	19.08	0.53	2070

Notes: Survey length refers to the median survey time measured in minutes (including speeders and laggers). High education refers to respondents with a university degree.

As survey participants were sampled via a double-opt-in online panel, this dataset cannot claim representativeness; quota sampling and weighting reduce but do not eliminate such biases^31^, and even probability samples now face declining response rates^32^. Point estimates should therefore be interpreted cautiously. Non-probability samples nonetheless remain widespread in the social sciences^33,34^ and can recover relationships between variables comparably to probability samples^31,35^. Furthermore, compared with other online samples like MTurk, Bilendi & respondi (the provider for all studies except Pre-Test 1) achieved high data quality with respondents passing multiple attention checks at rates similar to lab samples^36^.

### Ethics

The studies were approved by the ethics committee of the University of Mannheim to be conducted in the respective countries by Bilendi & respondi (EK Mannheim 38/2022 and 29/2024). Informed consent for participation and data sharing was retrieved from participants in written form before starting the survey.

### Materials

The main purpose of the studies was the construction and validation of new scales for radical and extremist political attitudes^29^ and the development of an implicit association test (IAT) to capture implicit extremist attitudes^17^. Furthermore, the data included additional motivators of political extremism according to the “3N” approach, i.e. the individual needs, the ideological narrative, and the social network^37^. For individual needs, the data includes anomia, authoritarian traits, material deprivation, socio-emotional disintegration, and political alienation. For ideological narrative and social networks, the data includes regional identifiers which allow the merging of relevant variables on the meso and macro level (e.g., share of voters by various parties, numbers of migrants, unemployment rate, etc.). Additionally, the data includes measures that make the expression of extremist attitudes more or less likely (grit, self-control, social desirability tendencies). Finally, it includes related concepts like conspiracy beliefs, moral grandstanding, and political violence justification which can be used to assess the potential consequences of holding various forms of radical, extremist, or fundamentalist attitudes. Table 2 displays the sample size, number of items, and Cronbach’s *α* for the indices measured across the pretests and main studies.

**Table 2:** Overview of available constructs by study.

Index	Source	N	No. items	*α*	*ω*	Pretest 1	Pretest 2	Pretest 3	Study 1	Study 2
General Extremism	Jungkunz *et al*. (2024)	15741	5	0.832	0.842	x^1^	x^6^	x^38^	x^23^	x^9^
Left-wing Radicalism	Jungkunz *et al*. (2024)	17521	6	0.777	0.840	x^8^	x^51^	x^52^	x^22^	x^10^
Right-wing Radicalism	Jungkunz *et al*. (2024)	17623	8	0.887	0.918	x^6^	x^48^	x^48^	x^23^	x^24^
Religious Fundamentalism	Altemeyer & Hunsberger (2004)	17530	6	0.822	0.870	x^6^	x^29^	x^29^	x^16^	x^10^
Implicit Political Extremism	Jungkunz *et al*. (2024b)	15116	1			x	x		x	x
Anomia	Srole (1956)	6103	4	0.715	0.750				x	
Anomia	Fischer & Kohr (1980)	6088	4	0.903	0.911				x	
Political Alienation	various	6125	17	0.930	0.945				x	
Authoritarianism	Oesterreich (2005)	6200	12	0.741	0.820				x	
Material Deprivation	Callan *et al*. (2011)	6017	5	0.654	0.745				x	
Social Desintegration	Heitmeyer *et al*. (2013)	5997	4	0.694	0.757				x	
MG: Prestige	Grubbs *et al*. (2019)	8310	6	0.776	0.841					x
MG: Dominance	Grubbs *et al*. (2019)	8269	4	0.906	0.907					x
Conspiracy Mentality	Bruder *et al*. (2013)	7462	5	0.881	0.909			x	x	
Self-control	Partsch & Danner (2020)	8329	4	0.605	0.726					x
Grit: perseverance	Duckworth *et al*. (2007)	8298	4	0.692	0.736					x
Grit: consistency	Duckworth *et al*. (2007)	8217	4	0.760	0.765					x
Social Desirability	Reynolds (1982)	8286	13	0.675	0.712					x
PVJ: general	Kalmoe (2014)	14216	5	0.789	0.850			x^18^	x^11^	x^5^
PVJ: specific	German Internet Panel	7367	6	0.924	0.942				x^4^	x^6^

N refers to the number of respondents who had valid responses on at least two items of the respective scale. Number of items indicates the number of items in the final scale. Superscripted numbers refer to the total number of available items for the respective construct in the respective study. Cronbach’s *α* and McDonald’s *ω* are calculated based on the items of the final scales. MG: moral grandstandig, PVJ: political violence justification.

#### General political extremism

The general political extremism scale (anti-democratic attitudes) is measured with a newly developed five-item battery (e.g., “Under some circumstances, a nondemocratic government can be preferable.” and “A concentration of power in one person guarantees order.”). For scale development purposes, the individual surveys also contain 34 additional items on general political extremism, which are not used for the final scale. Response options of all items ranged from fully disagree (1) to neither agree nor disagree (3) to fully agree (5). The psychometric validity and construct validity was assessed in previous work^29^, indicating very good fit according to conventional cut-off criteria^38^.

#### Left-wing radicalism

The left-wing radicalism scale is measured with a newly developed six-item battery (e.g., “A decent living is only possible with socialism.” and “Fascism shows the true face of capitalism.”). For scale development purposes, the individual surveys also contain 44 additional items on left-wing radicalism, which are not used for the final scale. Response options of all items ranged from fully disagree (1) to neither agree nor disagree (3) to fully agree (5). The psychometric validity and construct validity was assessed in previous work^29^, indicating very good fit according to conventional cut-off criteria^38^.

#### Right-wing radicalism

The right-wing radicalism scale is measured with a newly developed eight-item battery (e.g., “We should make sure we keep [nationality] pure and prevent nations mixing.” and “[Country] has become “too foreign” to a dangerous extent due to all the foreigners here.”). For scale development purposes, the individual surveys also contain 43 additional items on right-wing radicalism, which are not used for the final scale. Response options of all items ranged from fully disagree (1) to neither agree nor disagree (3) to fully agree (5). The psychometric validity and construct validity was assessed in previous work^29^, indicating very good fit according to conventional cut-off criteria^38^.

#### Religious fundamentalism

Religious fundamentalism was captured with the 12-item scale by Altemeyer and Hunsberger^8^ (e.g., “God has given humanity, a complete unfailing guide to happiness and salvation, which must be totally followed.” and “When you get right down to it, there are basically two kinds of people in the world: the righteous who will be rewarded by God; and the rest who will not.).” Furthermore, there are 13 additional items capturing other aspects of religious fundamentalism. Response options of all items ranged from fully disagree (1) to neither agree nor disagree (3) to fully agree (5).

#### Implicit political extremism

To assess implicit orientations toward political extremism, we administered a newly developed Implicit Association Test (IAT; see also^17,39^). The IAT measures the relative strength of automatic associations between target concepts and evaluative attributes by comparing participants’ response latencies during a series of categorization tasks^40^. Participants classify stimuli into paired categories using two response keys, with shorter reaction times indicating stronger underlying associative links between the combined concepts. Relative differences in response speed across compatible and incompatible category pairings provide an indirect measure of implicit attitudes that is less dependent on conscious self-report.

The IAT contrasted the target categories *democratic* and *undemocratic*, which were paired with positive and negative evaluative attributes. To operationalize the target concepts, we selected 15 terms reflecting democratic principles (e.g., “free elections”, “freedom of expression”, “rule of law”) and 13 terms representing undemocratic notions (e.g., “strong leader”, “right of the strongest”, “one-party rule”). Stimulus selection was guided by established conceptualizations of democracy and political extremism, while avoiding references to specific political actors, movements, or cultural contexts. Existing survey instruments on political extremism were additionally consulted to maximize content validity and facilitate comparisons between the implicit and explicit measures [see further^41^]. The evaluative stimuli were adapted from previous IAT applications^42^ and expanded with semantically related adjectives to better capture evaluations of democratic and anti-democratic concepts (e.g., “trustworthy” and “honorable” vs. “corrupt” and “untruthful”).

The task followed the standard seven-block IAT procedure. Participants first completed practice blocks in which they learned to categorize the target concepts and evaluative attributes separately. In the subsequent critical blocks, target and evaluative categories were combined into compatible (democratic & positive terms or undemocratic & negative words) and incompatible pairings (undemocratic & positive terms or democratic & negative words), with reaction times recorded for each trial. Implicit attitudes were quantified using the standardized D-score^43^, calculated from the latency difference between the two pairing conditions. Higher positive D-scores indicate relatively faster responses when extremist concepts are paired with positive attributes than when democratic concepts are paired with positive attributes, reflecting a comparatively stronger implicit preference for extremist over democratic concepts. The full procedure is explained in detail by Jungkunz *et al*. (2024)^17^.

#### Anomia

Anomia was captured with two separate batteries, a four-item scale by Fischer and Kohr^44^ (e.g., “Today, everything is changing so quickly that I often don’t know what to hold on to.”) and a four-item version of the scale developed by Srole^45^ (e.g., “No matter what some people say: The situation of ordinary people is not getting better, but worse”). Response options of all items ranged from fully disagree (1) to neither agree nor disagree (3) to fully agree (5).

#### Authoritarianism

Authoritarianism was captured with a twelve-item scale^46^. Respondents were presented with two opposing statements, one of which represented authoritarian traits and one of which contained open-minded traits (e.g., “People who are not on my side are against me [vs.] I can accept people who are not on my side”). Response options ranged from fully agree with the statement on the left-hand side (-2) to difficult to say (0) to fully agree with the statement on the right-hand side (+2).

#### Political alienation

Political alienation was captured with seventeen items. Given the lack of a pre-existing scale, the items were gathered from several existing surveys (e.g., the German Longitudinal Election Study, GLES) (e.g., “Most politicians are engaged in politics simply for their own personal advantage.” and “Most parties and politicians are corrupt.”). Response options of all items ranged from fully disagree (1) to neither agree nor disagree (3) to fully agree (5).

#### Material deprivation

For material deprivation, the data contains a five-item scale^47^ (e.g., “I feel deprived when I think about what I have compared to what other people like me have.” and “I feel resentful when I see how prosperous other people like me seem to be.”). Response options of all items ranged from fully disagree (1) to neither agree nor disagree (3) to fully agree (5).

#### Socio-emotional disintegration

For socio-emotional disintegration, the data contains four items used in the group-focused enmity studies [GMF,^48^] (e.g., “Social relationships are becoming increasingly unstable these days.” and “Finding real friends is becoming increasingly difficult today.”). Response options of all items ranged from fully disagree (1) to neither agree nor disagree (3) to fully agree (5).

#### Moral grandstanding

Moral grandstanding was captured with ten items^49,50^. These are grouped into two subdimensions: prestige-seeking grandstanding (e.g., “Even if expressing my views does not help anyone, it is important that I share them.”) and dominance-seeking grandstanding (e.g., “When I share my beliefs, I do so to show people who disagree with me that I am better than them.”). Response options of all items ranged from fully disagree (1) to neutral (3) to fully agree (7).

#### Conspiracy beliefs

For conspiracy beliefs, the data contains a five-item scale^51^ (e.g., “I think that government agencies closely monitor all citizens.” and “I think that events which superficially seem to lack a connection are often the result of secret activities.”). Respondents were asked to indicate how likely the respective statements are true. Response options of all items ranged from 0%—certainly not to 100%—definitely (with ten percentage point intervals in between).

#### Self-control

Self-control was measured with a five-item scale^52^ (e.g., “I usually make up my mind through careful reasoning.” and “When I am upset I often act without thinking.”). Response options of all items ranged from fully disagree (1) to neutral (3) to fully agree (5).

#### Grit

Grit was measured with an eight-item battery^53^. The battery captures two subdimensions of grit: consistency of interest (e.g., “I often set a goal but later choose to pursue a different one.”) and perseverance of effort (e.g., “I am a hard worker.”). Response options of all items ranged from fully applies to me (1) to applies to some degree to me (3) to does not apply at all to me (5).

#### Social desirability

The tendency for social desirability in responses to survey questions was captured with the 13-item Marlowe-Crowne Social Desirability Scale Form B^54,55^ (e.g., “I sometimes try to get even rather than forgive and forget.” and “I am always courteous, even to people who are disagreeable.”). Response options of all items ranged from agree (1) to disagree (2).

#### Political violence justification

Political violence justification was captured with two batteries. To measure a rather general support for political violence, the data include a five-item battery^56^ (e.g., “When politicians are damaging the country, citizens should send threats to scare them straight.”). Response options of all items ranged from fully disagree (1) to neither agree nor disagree (3) to fully agree (5). Furthermore, the data also contains six items that ask whether specific forms of political violence are acceptable^57^ (e.g., “Harassing a politician online in a way that makes them scared.”). Response options of all items ranged from never (1) to occationally (3) to always (5) acceptable behavior. Furthermore, there are 14 additional items capturing other aspects of political violence justification. All items are intended to be used separately (see further^58,59^).

#### Socio-demographics

All studies include additional items for political and religious identification, e.g., the left-right self-placement scale, past voting behavior and the intention to vote in the next election, religious affiliation, religiosity and support of religious organizations, as well as socio-demographic variables like age, sex, education, income, and occupation.

#### Regional identifiers

The data also include regional identifiers in Study 1 and Study 2. NUTS3-level identifiers are provided for France, Germany, Great Britain, Hungary, and the Netherlands. NUTS2-level identifiers are provided for Greece.

### Weights

The dataset includes three types of weights to ensure representativeness according to age, gender, education, and religion (and region in Germany). First, a correction weight adjusts for the oversampling of Muslims based on their actual population shares in the respective countries [see^60^]. In addition, socio-structural weights account for differences in the distribution of age, gender, and education, while, for Germany, an additional adjustment corrects for regional disparities between East and West Germany. These weights were constructed using country-specific population statistics from Eurostat^61,62^ and the Federal Statistical Office of Germany^63^. Specifically, base weights were derived from these sources, and survey-specific weights were computed as the ratio of population totals to observed sample counts. The resulting weights were then merged with the survey dataset and checked for completeness.

### Data processing

The data processing and harmonization were carried out systematically to ensure comparability across countries and studies and to prepare the datasets for analysis. Data import, management, cleaning, and transformation, were carried out in *R*^64^ via *tidyverse*^65^, *sjlabelled*^66^, and *sjmisc*^67^. The *survey*^68^ package was used to construct survey designs and compute weights, including raking procedures to adjust the sample to known population margins. Codebooks were created via codebookr^69^. Data quality checks were performed via careless^70^ and naniar^71^.

The following steps were implemented: Each dataset was loaded and, when needed, pre-processed for specific variables. If, for a study, the data were collected across different countries, the individual country datasets were merged to create a unified dataset before further processing. Several supplementary datasets were imported and prepared:

– Implicit Association Test (IAT) data: The D-Scores were calculated separately in *Stata*. This file was then merged with the main data sets [for the specific procedure, see^17^].

– Postal code and district data: German postal codes and district information were imported from official sources (German Federal Institute for Research on Building, Urban Affairs and Spatial Development (BBSR) and Deutsche Post) and harmonized for merging with survey data. For the final dataset, lower level regional identifiers were aggregated to NUTS3-level and NUTS2-level (only in Greece) regions in Study 1 and Study 2.

To construct the population weights, the following supplementary datasets were imported:

– Socio-demographic and population data: National population statistics were imported from Eurostat^61,62^ and the Federal Statistical Office^63^. Data included age, sex, education, and total population counts per country.

– Muslim population estimates: Pew Research Center data^60^ for the share of the Muslim population in European countries were combined with total population counts to calculate relative numbers and generate a variable which captures the share of Muslims within each country.

Afterwards, predefined sets of variables, including attitudes, socio-demographic measures, religious organization items, and country codes, were assigned harmonized value labels. Standardized coding schemes included:

– Attitude variables (1–5 or 1–7 scale)

– Socio-demographic binary indicators

– Country and country of origin codes

– Error and missing data labels

The merged survey datasets were recoded for consistency across countries and studies. Each respondent was assigned a unique ID and survey code. Attitudinal variables were recoded to harmonize naming and order across surveys. Age, sex, citizenship, birth country, educational attainment, occupation, household income, and household size were harmonized and transformed into comparable metrics across countries. Notably:

– Education levels were harmonized to ISCED 2011 categories.

– Income was converted to midpoints per category and to a numeric variable in euros, applying exchange rates for Hungary, Great Britain, and the USA. Voting behavior and party preference were recoded, handling missing values, inconsistencies, and country-specific differences.

In the data set of study 1, one duplicate was removed. The final, harmonized, and weighted datasets were saved for subsequent merging of all surveys and data analysis, ensuring reproducibility and consistency across studies.

## Data Records

The data is available at the GESIS Data Archive under the study identifier ZA9077 (“Radical and Extremist Attitudes in Europe”, https://doi.org/10.4232/1.14814)^72^. The data are categorized as category 0 “free access (with registration)”. Users have to create a GESIS account and agree with the general terms of services. The harmonized data set is provided in CSV (“ZA9077_v3-0-0.csv”), SPSS (“ZA9077_v3-0-0.sav”), and Stata format (“ZA9077_v3-0-0.dta”). Questionnaires (“ZA9077_q.pdf”), codebooks (“ZA9077_cod_all.pdf”), and method reports (“ZA9077_mr.pdf”) are available for all studies.

## Technical Validation

To assess the technical quality, reliability, and reusability of the dataset *Radical and extremist attitudes in Europe*, we conducted a series of validation procedures focusing on data completeness, attentiveness, internal consistency of measurement instruments, and the empirical validity of key constructs.

### Data completeness

Data completeness was assessed at both the respondent and item level. Across studies, only fully completed questionnaires were retained in the final datasets. Completion rates were high; for example, in Study 2, 9,103 respondents completed the survey out of eligible 14,613 starters, corresponding to a completion rate of 62.29%. Duplicate observations were identified and removed by the panel provider (e.g., N = 695 in Study 2 based on identical panel IDs), ensuring that each record represents a unique respondent. One duplicate in Study 1 was removed by the researchers in the process of preparing for publication. The overall percentage of missing data across variables was 1.6% in Pretest 1, 10.3% in Pre-Test 2, 9.5% in Pre-Test 3, 6.1% in Study 1, and 4.8% in Study 2. To calculate the shares, we removed columns with design-based missings due to filter, randomization, or country-specific questions.

### Attention check performance

Table 3 presents response quality checks for attentiveness, straightlining, extreme and midpoint responding, acquiescence, speeding, and long response times. Overall, there is little evidence for systematic bias. In some cases, we find higher extreme point responding (e.g., political violence justification or general extremism) but this is likely due to genuine disagreement with the quite radical or intensely worded items. The 100% extreme point responding rate for social desirability is the result of a two point scale where respondents can, by design, only answer with extreme endpoints. Across studies, we further tested for speeders (below 30% of median survey time, n_total_ = 154) and laggers (more than three times the median survey time, n_total_ = 912, see also Table 1). Again, there is no systematic evidence for speeding or lagging. Since there is no evidence that both affect survey quality per se, we created flag variables so that users can decide to include or remove them individually^73^.

**Table 3:** Response quality diagnostics by item battery.

Battery	Construct	Items	Response Options	% Straight- lined^1^	Mean IRV^2^	% Extreme Resp.^3^	% Midpoint Resp.^4^	Mean Resp. Level^5^
Pre-test 1	Right-wing radicalism	6	5	11.3	0.80	13.6	1.4	2.93
Pre-test 1	Religious fundamentalism	6	5	11.3	0.82	10.7	2.2	3.42
Pre-test 1	Left-wing radicalism	8	5	3.2	0.87	4.7	0.8	3.24
Pre-test 1	Political attitudes	10	6	1.4	1.31	4.3	—	3.35
Pre-test 2	Right-wing radicalism	48	5	2.4	1.08	16.1	3.2	2.49
Pre-test 2	Religious fundamentalism	27	5	1.9	1.33	19.9	2.7	2.94
Pre-test 2	Left-wing radicalism	49	5	1.8	1.09	4.9	3.5	3.02
Pre-test 2	General extremism	6	5	12.5	0.89	21.5	5.1	2.47
Pre-test 2	Right-wing radicalism	48	5	0.9	1.13	14.2	1.9	2.45
Pre-test 3	Religious fundamentalism	29	5	0.5	1.46	18.8	1.5	2.86
Pre-test 3	Left-wing radicalism	49	5	0.9	1.16	4.2	2.5	2.94
Pre-test 3	General extremism	38	5	0.8	1.31	16.4	2.3	2.52
Pre-test 3	Political violence justification	18	5	10.1	0.97	32.2	1.0	1.81
Pre-test 3	Conspiracy beliefs	5	11	7.9	1.70	6.6	3.4	5.85
Pre-test 3	Religious organizations	8	11	7.2	2.31	12.3	4.5	3.43
Study 1	General extremism	23	5	3.3	1.27	13.1	4.8	2.78
Study 1	Anomia	8	5	8.4	0.87	6.5	4.5	3.33
Study 1	Political alienation	17	5	4.3	1.00	9.4	5.4	3.42
Study 1	Authoritarianism	12	5	5.3	1.10	3.9	6.0	3.05
Study 1	Right-wing radicalism	23	5	4.3	1.08	11.8	5.0	2.63
Study 1	Religious fundamentalism	16	5	1.6	1.39	21.3	3.9	2.93
Study 1	Left-wing radicalism	22	5	3.3	1.07	5.3	6.0	2.92
Study 1	Political violence justification	11	5	5.7	1.04	48.9	3.9	1.99
Study 1	Conspiracy beliefs	5	11	7.9	1.63	6.0	3.6	6.70
Study 1	Material deprivation	5	5	14.8	0.97	13.3	14.6	2.84
Study 1	Socio-emotional disintegration	4	5	19.5	0.70	7.9	8.9	3.59
Study 1	Religious organizations	8	5	5.0	0.94	2.0	0.9	1.91
Study 2	Right-wing radicalism	24	5	0.8	1.07	10.6	2.3	2.73
Study 2	Religious fundamentalism	10	5	3.3	1.22	24.0	6.4	2.85
Study 2	Left-wing radicalism	10	5	3.7	1.01	7.0	5.8	2.90
Study 2	General extremism	9	5	2.7	1.15	17.2	3.8	2.49
Study 2	Grit	8	5	3.4	1.04	4.5	4.0	3.22
Study 2	Self-control	4	5	5.9	1.12	6.4	3.2	3.31
Study 2	Moral grandstanding	10	7	5.9	1.41	5.0	6.1	3.80
Study 2	Political violence justification	5	5	15.2	1.25	25.4	6.2	2.95
Study 2	Social desirability	13	2	2.5	0.48	100.0	-	1.54
Study 2	Anti-semitism	4	5	8.5	1.16	13.2	8.4	3.15
Study 2	Religious organizations	10	5	13.9	0.92	18.1	9.3	2.16

^1^For each respondent, the longest run of identical consecutive answers within the battery was computed (see^70,74^). A respondent was flagged if this run covered ≥80% of the battery’s items. The column reports the percentage of respondents flagged. Should be viewed with caution when the number of items in a battery is low as it may be unreliable. ^2^IRV = intra-individual response variability across a battery’s items (see^70,74^). Lower values indicate more item nondifferentiation (flatter response patterns). ^3^For each respondent, the proportion of the battery’s items answered at either scale endpoint (minimum or maximum value) was computed. A respondent was flagged if this proportion was ≥0.8 (i.e., ≥80% of items answered at an extreme). The column reports the percentage of respondents flagged. ^4^For each respondent, the proportion of the battery’s items answered at the midpoint was computed. A respondent was flagged if this proportion was ≥0.8 (i.e., ≥80% of items answered at the midpoint). The column reports the percentage of respondents flagged. Only defined for scales with a true midpoint (odd number of scale points); shown as ’-’ otherwise. ^5^Each respondent’s mean raw response across the battery’s items, averaged across respondents. Mean response level is used as an acquiescence proxy. Where a battery lacks reverse-keyed items, this value conflates genuine attitude/trait level with agreement tendency and should be read as an approximate indicator.

### Scale validations

To assess the internal consistency of the attitudinal measures, Cronbach’s *α* and McDonald’s *ω* coefficients were calculated for all multi-item scales (see Table 2). Across studies, most scales show acceptable to high levels of internal consistency.

Furthermore, we provide CFA (Table 4) and measurement invariance results (Table 5) for all scales that are available in the dataset. First, according to the CFA results from the pooled data, all scales show good or decent fit. Secondly, almost all scales achieve metric invariance or are only slightly above the threshold for *Δ*CFI but still under the threshold for *Δ*RMSEA. Thus, effect sizes can be compared meaningfully across countries. Scalar invariance is achieved in most cases only after freeing some intercepts (mostly one or two), indicating that these scales achieved partial scalar invariance (except authoritarianism) which is still good enough to compare means across countries^75^.

**Table 4:** Confirmatory factor analyses results for available scales.

Index	*k*	*χ*^2^	*df*	*p*	RMSEA	SRMR	CFI
General extremism	5	62.16	5	< .001	0.028	0.010	0.996
Left-wing radicalism	6	316.43	8	< .001	0.052	0.024	0.972
Right-wing radicalism	8	836.16	13	< .001	0.066	0.029	0.978
Religious fundamentalism	6	319.01	8	< .001	0.052	0.026	0.983
Anomia^1^	4	61.31	2	< .001	0.070	0.019	0.981
Anomia^2^	4	20.57	2	< .001	0.039	0.007	0.997
Political alienation	17	2409.00	118	< .001	0.056	0.036	0.938
Authoritarianism	12	576.55	25	< .001	0.060	0.039	0.944
Material deprivation	5	184.05	4	< .001	0.086	0.033	0.961
Social desintegration	4	4.71	1	0.030	0.025	0.005	0.999
Conspiracy mentality	5	70.79	4	< .001	0.053	0.013	0.992
Moral grandstanding	10	399.42	34	< .001	0.036	0.026	0.983
Self-control	4	2.23	2	0.328	0.004	0.005	1.000
Grit	8	283.68	19	< .001	0.041	0.031	0.972
Social desirability	13	534.41	64	< .001	0.033	0.048	0.971

Note. *k* = number of indicators. ^1^Srole (1956); ^2^Fischer & Kohr (1980).

**Table 5:** Measurement invariance tests across countries.

Index	Model	RMSEA	SRMR	CFI	*Δ*CFI	*Δ*RMSEA
General Extremism	Configural	0.037	0.013	0.993	—	—
	Metric	0.037	0.027	0.988	−0.005	0.001
	Scalar	0.073	0.048	0.937	−0.051	0.036
	Scalar (partial, *k* = 2 freed)	0.041	0.030	0.983	−0.006	0.004
Left-wing Radicalism	Configural	0.068	0.032	0.956	—	—
	Metric	0.063	0.043	0.945	−0.010	−0.005
	Scalar	0.077	0.056	0.894	−0.051	0.014
	Scalar (partial, *k* = 3 freed)	0.065	0.045	0.938	−0.007	0.002
Right-wing Radicalism	Configural	0.079	0.035	0.969	—	—
	Metric	0.081	0.055	0.959	−0.010	0.002
	Scalar	0.094	0.064	0.933	−0.026	0.013
	Scalar (partial, *k* = 2 freed)	0.084	0.058	0.951	−0.007	0.003
Religious Fundamentalism	Configural	0.059	0.030	0.978	—	—
	Metric	0.061	0.050	0.965	−0.013	0.002
	Scalar	0.070	0.056	0.941	−0.023	0.009
	Scalar (partial, *k* = 2 freed)	0.065	0.052	0.955	−0.010	0.004
Anomia^1^	Configural	0.080	0.022	0.974	—	—
	Metric	0.063	0.028	0.968	−0.006	−0.017
	Scalar	0.076	0.042	0.931	−0.037	0.013
	Scalar (partial, *k* = 2 freed)	0.065	0.032	0.961	−0.007	0.002
Anomia^2^	Configural	0.038	0.007	0.998	—	—
	Metric	0.034	0.016	0.996	−0.001	−0.004
	Scalar	0.039	0.019	0.992	−0.004	0.005
	Scalar (partial)	0.039	0.019	0.992	−0.004	0.005
Political Alienation	Configural	0.059	0.039	0.932	—	—
	Metric	0.058	0.052	0.927	−0.006	−0.000
	Scalar	0.064	0.058	0.906	−0.020	0.005
	Scalar (partial, *k* = 2 freed)	0.060	0.055	0.917	−0.010	0.002
Authoritarianism	Configural	0.065	0.046	0.918	—	—
	Metric	0.064	0.058	0.902	−0.016	−0.000
	Scalar	0.071	0.064	0.863	−0.039	0.006
	Scalar (partial, *k* = 3 freed)	0.065	0.059	0.891	−0.011	0.001
Material Deprivation	Configural	0.083	0.032	0.964	—	—
	Metric	0.082	0.059	0.943	−0.022	−0.002
	Scalar	0.092	0.066	0.905	−0.038	0.011
	Scalar (partial, *k* = 2 freed)	0.081	0.060	0.938	−0.004	−0.001
Social Desintegration	Configural	0.019	0.005	0.999	—	—
	Metric	0.018	0.012	0.998	−0.001	−0.001
	Scalar	0.031	0.017	0.993	−0.006	0.013
	Scalar (partial)	0.031	0.017	0.993	−0.006	0.013
Conspiracy Mentality	Configural	0.057	0.015	0.991	—	—
	Metric	0.054	0.029	0.987	−0.004	−0.003
	Scalar	0.077	0.042	0.963	−0.024	0.023
	Scalar (partial, *k* = 2 freed)	0.054	0.031	0.984	−0.003	0.000
Moral Grandstanding	Configural	0.049	0.048	0.969	—	—
	Metric	0.048	0.054	0.965	−0.004	−0.001
	Scalar	0.069	0.069	0.918	−0.047	0.020
	Scalar (partial, *k* = 3 freed)	0.053	0.056	0.953	−0.012	0.005
Self-control	Configural	0.033	0.022	0.992	—	—
	Metric	0.033	0.025	0.991	−0.002	−0.000
	Scalar	0.042	0.030	0.978	−0.012	0.009
	Scalar (partial, *k* = 1 freed)	0.039	0.028	0.984	−0.007	0.006
Grit	Configural	0.041	0.033	0.973	—	—
	Metric	0.041	0.038	0.966	−0.006	−0.000
	Scalar	0.049	0.044	0.942	−0.024	0.008
	Scalar (partial, *k* = 3 freed)	0.043	0.040	0.959	−0.007	0.002
Social Desirability	Configural	0.033	0.058	0.973	—	—
	Metric	0.036	0.065	0.963	−0.010	0.003
	Scalar	0.036	0.065	0.963	−0.000	0.000
	Scalar (partial)	0.036	0.065	0.963	−0.000	0.000

Note.*Δ* = change relative to the metric model (configural for metric row). Non-invariance per Chen (2007): *Δ*CFI < − . 010 or *Δ*RMSEA ≥. 015. Freed intercepts (or thresholds for social desirability) in partial scalar model determined iteratively by largest score test. ^1^Srole (1956); ^2^Fischer & Kohr (1980).

### Construct validity of radicalism, extremism, and fundamentalism

**Fig. 1:**
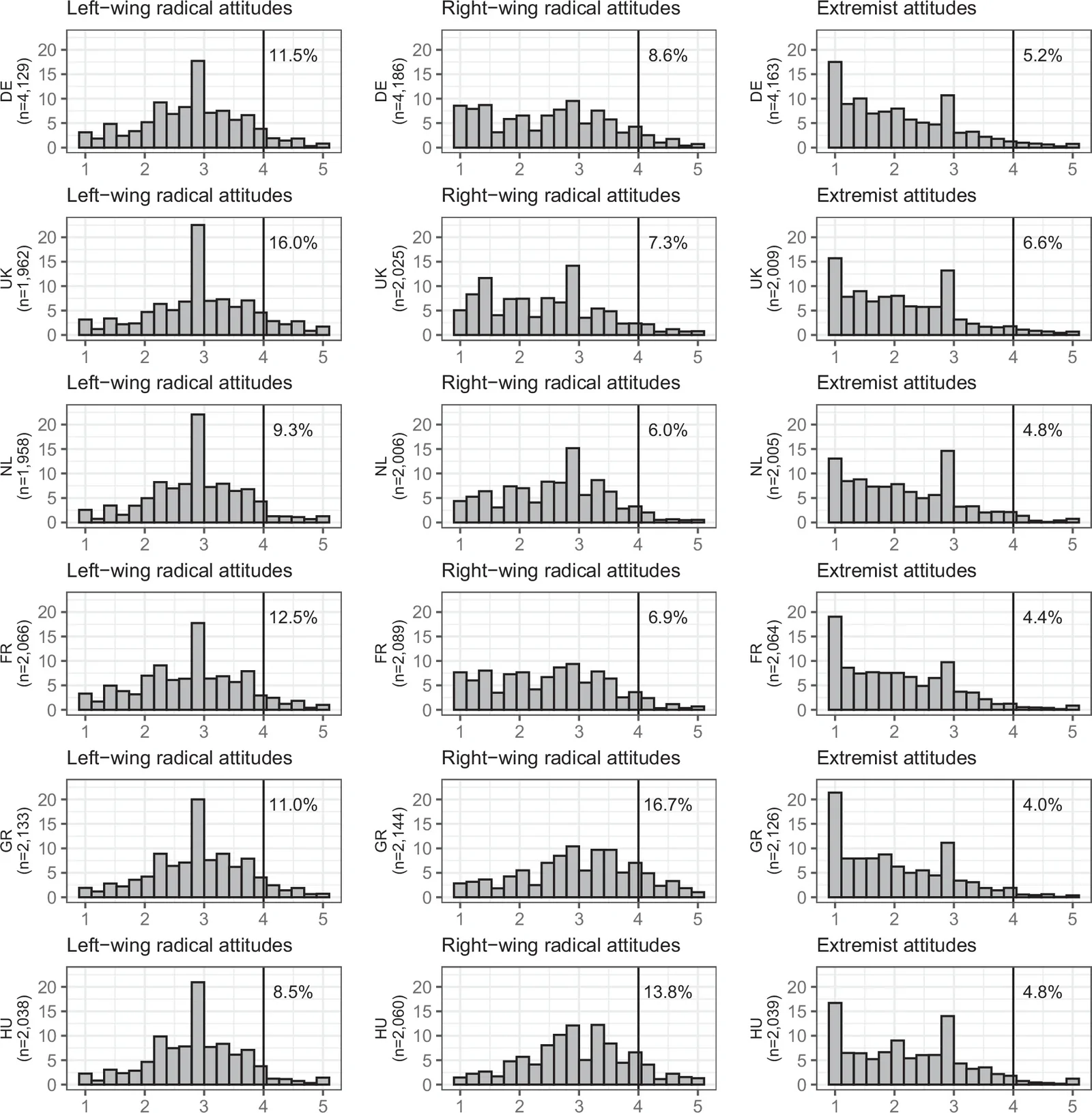
Distributions of radicalism and extremism scales by country. Shown are distributions of indices of left- and right-wing radical and general extremist attitudes by country in percentages. Respondents are categorized as radical or extremist if they scored four or higher on the respective indices, meaning they (strongly) agreed with the respective items on average. Percentages refer to the share of respondents who (strongly) agreed with the items of the respective scale on average. Data: Study 1 and Study 2.

To validate the measurement of radicalism, extremism, and religious fundamentalism, we examined correlations between key constructs and theoretically related variables (see Table 6). The observed relationships are consistent with theoretical expectations, supporting the construct validity of the measures. We first explored the distribution of key variables, including the radicalism and extremism scales (see Fig. 1). Across all countries, the distributions are characterized by a pronounced left-skew, with most respondents reporting low levels of radical and extremist attitudes and progressively fewer respondents at higher scale values. This pattern is theoretically expected, given that only a minority of the population typically holds extremist attitudes^6^. Importantly, the presence of a non-negligible proportion of respondents at higher scale values indicates that the measures capture meaningful variation and are not dominated by floor effects. At the same time, the absence of clustering at the upper bound suggests that ceiling effects are negligible. Differences in the upper tail of the distributions across countries further point to substantively meaningful cross-national variation, e.g., high levels of right-wing radical attitudes in Greece (16.7%) and Hungary (13.8%) relative to other countries (<9%).

**Table 6:** Correlations between predictors and radicalism and extremism scales.

Predictor	LWR	RWR	GEX	REF
Anomia	0.31	0.33	0.34	0.20
Authoritarianism	0.00	0.30	0.22	0.13
Conspiracy beliefs	0.34	0.31	0.17	0.16
Self-control	0.21	0.20	0.21	0.06
Grit	0.23	0.24	0.27	0.12
Material deprivation	0.31	0.41	0.44	0.22
Socio-emotional disintegration	0.15	0.10	0.01	–0.02
Moral grandstanding: prestige-seeking	0.21	0.18	0.13	0.11
Moral grandstanding: dominance-seeking	0.29	0.36	0.55	0.36
Political alienation	0.28	0.25	0.05	–0.05
Political violence justification: general	0.30	0.47	0.43	0.29
Political violence justification: specific	0.32	0.44	0.56	0.35

Shown are weighted Pearson r values to correct for Muslim oversampling. LWR: left-wing radicalism, RWR: right-wing radicalism, GEX: general extremism, REF: religious fundamentalism. Data: Study 1 for anomia, authoritarianism, conspiracy beliefs, material deprivation, socio-emotional disintegration, political alienation, and political violence justification; Study 2 for self-control, grit, moral grandstanding, and political violence justification.

**Fig. 2:**
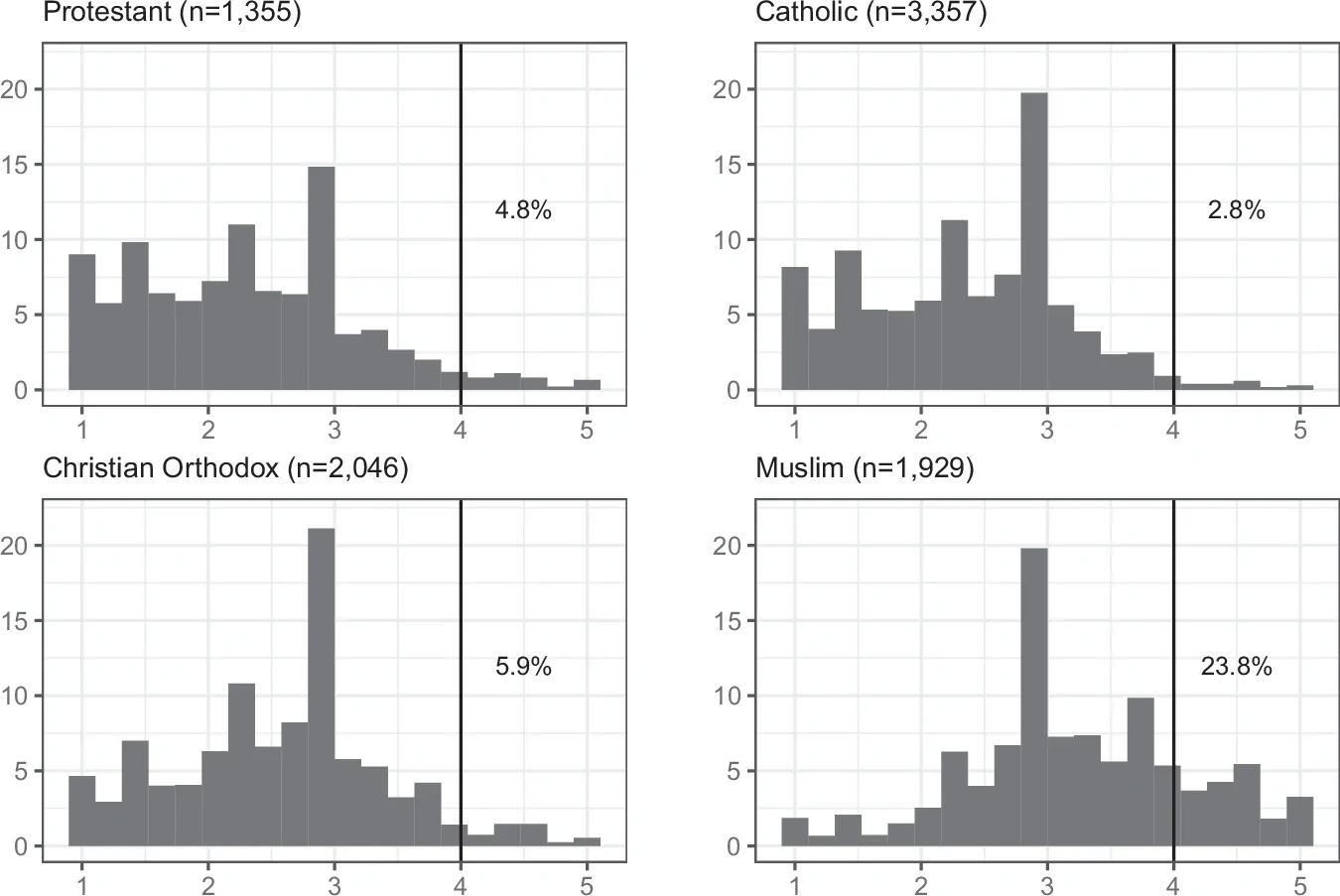
Distributions of religious fundamentalism across all countries. Shown are distributions of the religious fundamentalism index^8^ by religion in percentages. The y-axis indicates percentages. Percentages to the right of the vertical line refer to the share of respondents who (strongly) agreed with the items of the respective scale on average. Data: Study 1 and Study 2.

Furthermore, the data also show substantial variation of religious fundamentalism across countries and/or religious affiliations. Due to the oversampling of Muslims, it is possible to draw more precise estimates for religious minorities, which are generally rather underrepresented in surveys. Fig. 2 displays the distribution of religious fundamentalism as measured by the Altemeyer and Hunsberger^8^ scale across all countries. The share of religious fundamentalists ranges from 2.8% among Catholics, to 4.8% among Protestants, 5.9% among Christian Orthodox, and 23.8% among Muslims.

**Fig. 3:**
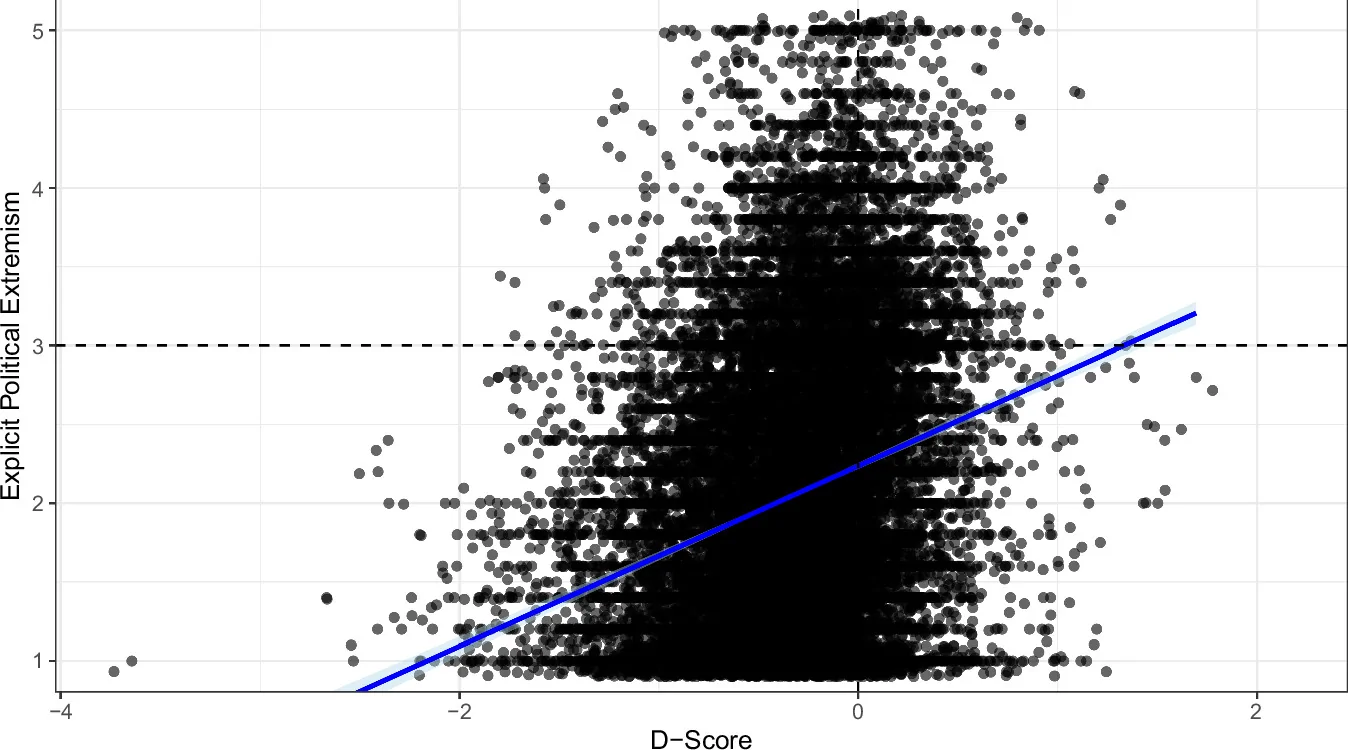
Scatterplot of D-score and explicit political extremism. Scatterplot based on values on the general political extremism scale (y-axis) and the D-Score for implicit extremist attitudes (x-axis). The dashed lines indicate the midpoint of the scales. Data: Study 1 and Study 2, N =13,061.

The dataset includes an Implicit Association Test (IAT) to measure implicit political extremism. The IAT was implemented following established procedures, and D-scores were computed based on standardized algorithms^76,77^. The validation of the IAT can be reviewed in earlier work^17^. The implicit association test returns a standardized D-Score, which ranges from a preference of democracy over extremism (negative values) to a preference of extremism over democracy (positive values). Since there may be a variety of reasons why respondents do or do not state their true attitudes, e.g., non-awareness, social desirability, the data allow for a contrast between implicit and explicit measures. Fig. 3 displays the values of the D-Score and the index of general political extremism. Although both measures are related (r = 0.268), they also capture different parts of the population. The data points show substantial variability, but the upward trend suggests that individuals with stronger implicit biases also tend to report higher levels of explicit extremism. This is in line with other findings which show that correlations between IATs and explicit measures can vary substantially between 0.10 and 0.70^41^. In most cases, correlations are small to moderate, but they are generally rather low for topics with high social sensitivity, as in the case of political extremism^78,79^.

Table 6 presents the correlates of political radicalism, extremism, and religious fundamentalism with various potential predictors and consequences. Overall, the concepts correlate moderately in most cases, with similarities across radicalism, extremism, and fundamentalism. However, there are also some important differences which can be studied cross-nationally or among various subgroups of the populations (see also^30^ for a detailed analysis of commonalities and differences between radicals, extremists, and fundamentalists).

**Fig. 4:**
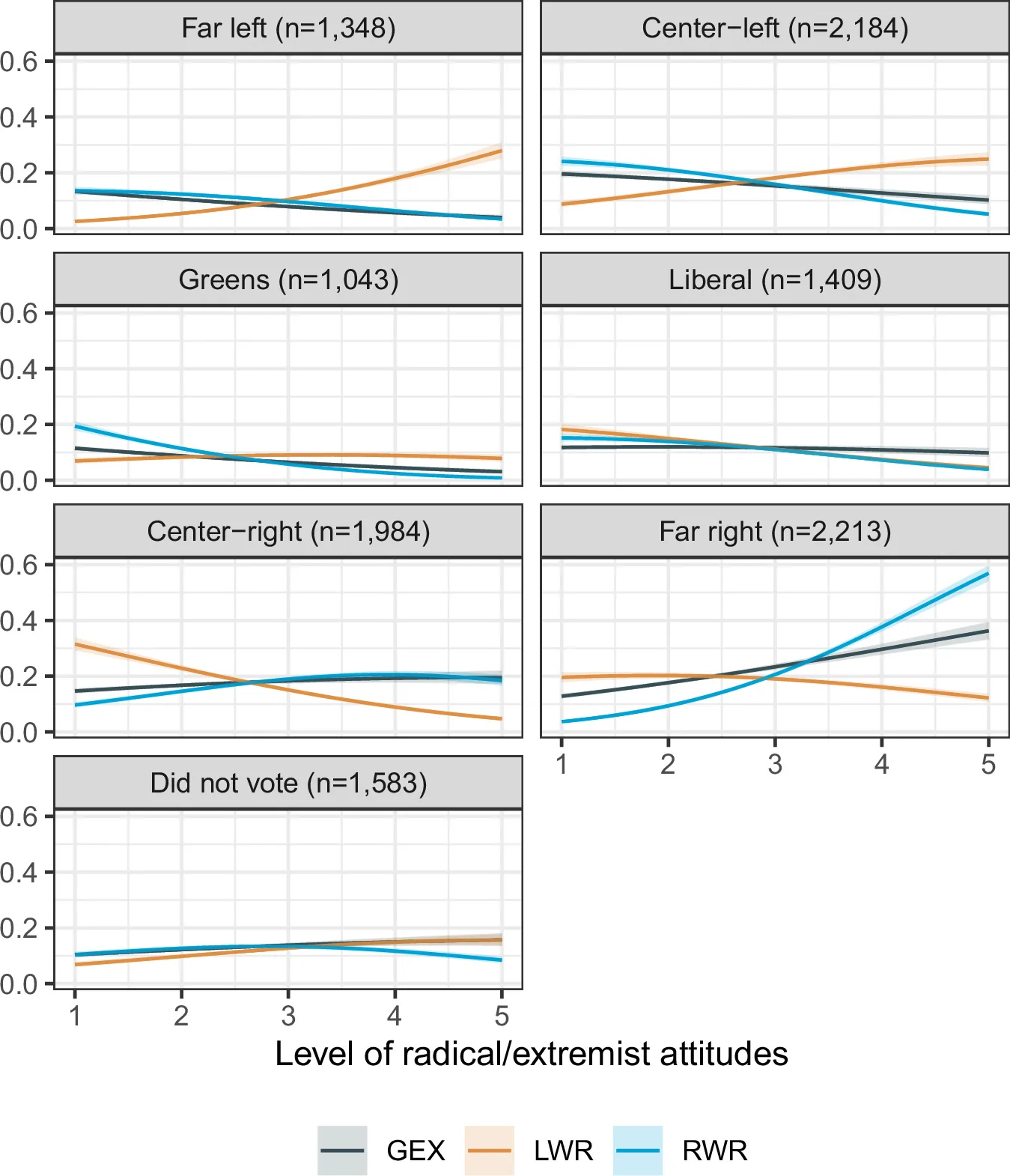
Predictions of vote choice by radicalism and extremism. Predicted probabilities to vote for the respective party families by values of the left- and right-wing radicalism and general political extremism indices with 95% confidence intervals. Results are weighted to correct for Muslim oversampling. LWR: left-wing radicalism, RWR: right-wing radicalism, GEX: general extremism. Data: Study 1 and Study 2.

Finally, the attitudinal measures predict voting for a particular political party. Figure 4 shows the vote propensity for various party families by political radicalism and extremism scale. As expected, there is a higher likelihood to vote for far-left parties among respondents with a higher degree of left-wing radicalism and a higher propensity to vote for the far right among respondents with a higher degree of right-wing radicalism. Higher general extremism is also associated with a higher likelihood of voting for the far right but not the far left. Future research can study these insights and relationships within the individual countries and respective subgroups, or include moderators that may impact the relationship between attitudes and behavior.

## Data Availability

The data is available at the GESIS Data Archive under the study identifier ZA9077 (“Radical and Extremist Attitudes in Europe”, https://doi.org/10.4232/1.14814)^72^. Users have to create a free GESIS account and agree with the general terms of services. The dataset is curated and published in collaboration with the Data Portal for Racism and Right-Wing Extremism Research (DP-R∣EX), a dedicated research data infrastructure for Germany that facilitates data reusability in line with the FAIR Principles^80^.

## Code availability

The code to create all figures and Tables 3 and 6 in this paper is available in the corresponding author’s Harvard Dataverse (https://doi.org/10.7910/DVN/4ES5FB).

## References

[1] Levitsky, S. & Ziblatt, D.* How Democracies Die* (Crown, New York, 2018).

[2] Norris, P. & Inglehart, R.* Cultural Backlash: Trump, Brexit, and Authoritarian Populism* (Cambridge University Press, Cambridge, 2019).

[3] Puddington, A.* Breaking Down Democracy: Goals, Strategies, and Methods of Modern Authoritarians* (Freedom House, Washington, 2017).

[4] Repucci, S.* Freedom in the World 2020: A Leaderless Struggle for Democracy* (Freedom House, Washington, 2020).

[5] Backes, U. Meaning and Forms of Political Extremism in Past and Present. *Central European Political Studies Review* **4**, 242–262 (2007).

[6] Jungkunz, S.* The Nature and Origins of Political Extremism In Germany and Beyond* (Palgrave Macmillan, Cham, 2022).

[7] Altemeyer, B. & Hunsberger, B. Authoritarianism, Religious Fundamentalism, Quest, and Prejudice. *International Journal for the Psychology of Religion* **2**, 113–133 (1992).

[8] Altemeyer, B. & Hunsberger, B. A Revised Religious Fundamentalism Scale: The Short and Sweet of It. *International Journal for the Psychology of Religion* **14**, 47–54 10.1207/s15327582ijpr1401_4 (2004).

[9] Mudde, C. The Populist Radical Right: A Pathological Normalcy. *West European Politics* **33**, 1167–1186 10.1080/01402382.2010.508901 (2010).

[10] Zinchenko, Y. P. Extremism from the perspective of a system approach. *Psychology in Russia: State of the Art* **7**, 23–33 10.11621/pir.2014.0103 (2014).

[11] Pérez, E. O.* Unspoken Politics. Implicit Attitudes and Political Thinking* (Cambridge University Press, Cambridge, 2016).

[12] Valentim, V. Political Stigma and Preference Falsification: Theory and Observational Evidence. *The Journal of Politics* **86**, 1382–1402 10.1086/729966 (2024).

[13] Burdein, I., Lodge, M. & Taber, C. Experiments on the Automaticity of Political Beliefs and Attitudes. *Political Psychology* **27**, 359–371 10.1111/j.1467-9221.2006.00504.x (2006).

[14] Erisen, C., Lodge, M. & Taber, C. S. Affective Contagion in Effortful Political Thinking. *Political Psychology* **35**, 187–206 10.1111/j.1467-9221.2012.00937.x (2014).

[15] Lodge, M., Taber, C. & Verhulst, B. Conscious and Unconscious Information Processing with Implications for Experimental Political Science. In Druckman, J. N., Green, D. P., Kuklinski, J. H. & Lupia, A. (eds.) *Cambridge Handbook of Experimental Political Science*, 155–170 (Cambridge University Press, Cambridge, 2011).

[16] Taber, C. S. & Lodge, M. The Illusion of Choice in Democratic Politics: The Unconscious Impact of Motivated Political Reasoning: Illusion of Choice in Democratic Politics. *Political Psychology* **37**, 61–85 10.1111/pops.12321 (2016).

[17] Jungkunz, S., Helbling, M. & Isani, M. Measuring Implicit Political Extremism through Implicit Association Tests. *Public Opinion Quarterly* **88**, 175–192 10.1093/poq/nfad061 (2024).

[18] Jungkunz, S. Towards a Measurement of Extreme Left-Wing Attitudes. *German Politics* **28**, 101–122 10.1080/09644008.2018.1484906 (2019).

[19] Bauer, P. C., Barberá, P., Ackermann, K. & Venetz, A. Is the Left-Right Scale a Valid Measure of Ideology? *Political Behavior* **39**, 553–583 10.1007/s11109-016-9368-2 (2017).

[20] Tourangeau, R. & Yan, T. Sensitive Questions in Surveys. *Psychological Bulletin* **133**, 859–883 10.1037/0033-2909.133.5.859 (2007). 17723033

[21] Conover, P. J. & Feldman, S. The Origins and Meaning of Liberal/Conservative Self-Identifications. *American Journal of Political Science* **25**, 617–645 (1981).

[22] Neundorf, A. Growing up on Different Sides of the Wall – A Quasi-Experimental Test: Applying the Left-Right Dimension to the German Mass Public. *German Politics* **18**, 201–225 10.1080/09644000902870834 (2009).

[23] de Vries, C. E., Hakhverdian, A. & Lancee, B. The Dynamics of Voters’ Left/Right Identification: The Role of Economic and Cultural Attitudes. *Political Science Research and Methods* **1**, 223–238 10.1017/psrm.2013.4 (2013).

[24] Kiess, J. & Decker, O. Comparing right-wing extremist attitudes: lack of research or lack of theory? In Kiess, J., Brähler, E. & Decker, O. (eds.) *German Perspectives on Right-Wing Extremism. Challenges for comparative analysis*, 11–30 (Routledge, Abingdon, Oxon, 2016).

[25] Cattacin, S., Gerber, B., Sardi, M. & Wegener, R.* Monitoring rightwing extremist attitudes, xenophobia and misanthropy in Switzerland*. Research report - PNR 40+, Sociograph - Sociological Research. Study No 1 of the Department of Sociology (University of Geneva, Geneva, 2006).

[26] Flecker, J. *et al*. (eds.) *SIREN: Socio-Economic Change, Individual Reactions and the Appeal of the Extreme Right* (Commission of the European Communities, Directorate-General, Luxembourg, 2006).

[27] Manzoni, P. *et al*. *Einflussfaktoren extremistischer Einstellungen unter Jugendlichen in der Schweiz* (ZHAW Zürcher Hochschule für Angewandte Wissenschaften, Zürich, 2019).

[28] Zick, A. *et al*. The Syndrome of Group-Focused Enmity: The Interrelation of Prejudices Tested with Multiple Cross-Sectional and Panel Data. *Journal of Social Issues* **64**, 363–383 (2008).

[29] Jungkunz, S., Helbling, M. & Osenbrügge, N. Measuring political radicalism and extremism in surveys: three new scales. *PLOS One* **19**, e0300661 10.1371/journal.pone.0300661 (2024). PMC11078353 38718012

[30] Helbling, M., Jungkunz, S. & Osenbrügge, N. A comparison of individual needs and support of violence among radicals, extremists and fundamentalists in western europe. *Humanities and Social Sciences Communications* **12** (2025).

[31] Rohr, B., Silber, H. & Felderer, B. Comparing the Accuracy of Univariate, Bivariate, and Multivariate Estimates across Probability and Nonprobability Surveys with Population Benchmarks. *Sociological Methodology* **55**, 121–154 10.1177/00811750241280963 (2025).

[32] de Leeuw, E., Hox, J. & Luiten, A. International Nonresponse Trends across Countries and Years: An analysis of 36 years of Labour Force Survey data. *Survey Insights: Methods from the Field* 10.13094/SMIF-2018-00008 (2018).

[33] Cornesse, C. *et al*. A Review of Conceptual Approaches and Empirical Evidence on Probability and Nonprobability Sample Survey Research. *Journal of Survey Statistics and Methodology* **8**, 4–36 10.1093/jssam/smz041 (2020).

[34] Freese, J. & Jin, O. Online Nonprobability Samples. *Annual Review of Sociology* **51**, 109–128 10.1146/annurev-soc-090524-043117 (2025).

[35] Pasek, J. & Krosnick, J. A. Relations Between Variables and Trends Over Time in RDD Telephone and Nonprobability Sample Internet Surveys. *Journal of Survey Statistics and Methodology* **8**, 37–61 10.1093/jssam/smz059 (2020).

[36] Celebi, C. *et al*. Mission Possible: The Collection of High-Quality Data. *CESifo Working Paper*10.65864/q5ekls6q5u (2026).

[37] Kruglanski, A. W., Bélanger, J. J. & Gunaratna, R. *The Three Pillars of Radicalization: Needs, Narratives, and Networks* (Oxford University Press, New York, 2019).

[38] Hu, L. & Bentler, P. M. Cutoff Criteria for Fit Indexes in Covariance Structure Analysis. *Structural Equation Modeling: A Multidisciplinary Journal* **6**, 1–55 10.1080/10705519909540118 (1999).

[39] Jungkunz, S. & Helbling, M. Beyond self-reports: Exploring implicit biases in right-wing political extremism. In Fernandes, J. M. & Martínez-Cantó, J. (eds.) *Democracy at the Crossroads* — *Challenges for Governance and Representation*, 589–606 (Routledge, London, 2026).

[40] Greenwald, A. G., McGhee, D. E. & Schwartz, J. L. K. Measuring Individual Differences in Implicit Cognition: The Implicit Association Test. *Journal of Personality and Social Psychology* **74**, 1464–1480 (1998). 10.1037//0022-3514.74.6.1464 9654756

[41] Nosek, B. A. Moderators of the Relationship Between Implicit and Explicit Evaluation. *Journal of Experimental Psychology: General* **134**, 565–584 10.1037/0096-3445.134.4.565 (2005). PMC1440676 16316292

[42] Carpenter, T. P. *et al*. Survey-software implicit association tests: A methodological and empirical analysis. *Behavior Research Methods* **51**, 2194–2208 10.3758/s13428-019-01293-3 (2019). 31515742

[43] Greenwald, A. G., Nosek, B. A. & Banaji, M. R. Understanding and Using the Implicit Association Test: I. An Improved Scoring Algorithm. *Journal of Personality and Social Psychology* **85**, 197–216 10.1037/0022-3514.85.2.197 (2003). 12916565

[44] Fischer, A. & Kohr, H.-U.* Politisches Verhalten und empirische Sozialforschung: Leistung und Grenzen von Befragungsinstrumenten* (Juventa, München, 1980).

[45] Srole, L. Social Integration and Certain Corollaries: An Exploratory Study. *American Sociological Review* **21**, 706–716 (1956).

[46] Oesterreich, D. Flight into Security: A New Approach and Measure of the Authoritarian Personality. *Political Psychology* **26**, 275–297 (2005).

[47] Callan, M. J., Shead, N. W. & Olson, J. M. Personal Relative Deprivation, Delay Discounting, and Gambling. *Journal of Personality and Social Psychology* **101**, 955–973 10.1037/a0024778 (2011). 21875231

[48] Heitmeyer, W. *et al*. Group-oriented animosity against people (GMF-Survey 2011), 10.4232/1.11807. Version Number: 1.0.0 Type: dataset (2013).

[49] Grubbs, J. B., Warmke, B., Tosi, J., James, A. S. & Campbell, W. K. Moral grandstanding in public discourse: Status-seeking motives as a potential explanatory mechanism in predicting conflict. *PLOS ONE* **14**, e0223749 10.1371/journal.pone.0223749 (2019). PMC6795490 31618235

[50] Jungkunz, S. The age of virtue signaling: Moral grandstanding as competitive display among young men. *Political Psychology* **47**, e70132 10.1111/pops.70132 (2026).

[51] Bruder, M., Haffke, P., Neave, N., Nouripanah, N. & Imhoff, R. Measuring Individual Differences in Generic Beliefs in Conspiracy Theories Across Cultures: Conspiracy Mentality Questionnaire. *Frontiers in Psychology* **4**, 10.3389/fpsyg.2013.00225 (2013). PMC3639408 23641227

[52] Partsch, M. V. & Danner, D. Measuring Self-Control in International Large-Scale Surveys: Development and Validation of a Four-Item Scale in English, French, German, Japanese, Polish, and Spanish. *European Journal of Psychological Assessment* **37**, 409–418 10.1027/1015-5759/a000618 (2021).

[53] Duckworth, A. L., Peterson, C., Matthews, M. D. & Kelly, D. R. Grit: Perseverance and passion for long-term goals. *Journal of Personality and Social Psychology* **92**, 1087–1101 10.1037/0022-3514.92.6.1087 (2007). 17547490

[54] Bursztyn, L., Haaland, I., Röver, N. & Roth, C. The Social Desirability Atlas. *NBER Working Paper*** 33920**, 10.3386/w33920 (2025).

[55] Reynolds, W. M. Development of reliable and valid short forms of the marlowe-crowne social desirability scale. *Journal of Clinical Psychology* **38**, 119–125 (1982).

[56] Kalmoe, N. P. From Fistfights to Firefights: Trait Aggression and Support for State Violence. *Political Behavior* **35**, 311–330 10.1007/s11109-012-9195-z (2013).

[57] German Internet Panel, University of Mannheim. German Internet Panel, wave 60 (July 2022), 10.4232/1.14364 (2024).

[58] Kalmoe, N. P. & Mason, L.* Radical American Partisanship. Mapping Violent Hostility, Its Causes, and the Consequences for Democracy* (University of Chicago Press, Chicago, 2022).

[59] Westwood, S., Grimmer, J., Tyler, M. & Nall, C. Current research overstates American support for political violence. *Proceedings of the National Academy of Sciences* **119**, e2116870119 10.1073/pnas.2116870119 (2022). PMC8944847 35302889

[60] Pew Research Center. 5 facts about the Muslim population in Europe. https://www.pewresearch.org/short-reads/2017/11/29/5-facts-about-the-muslim-population-in-europe/. Accessed: 2025-01-17, 17:09:00 (2017).

[61] Eurostat. Edat_lfs_9901 dataset from Eurostat database, 10.2908/EDAT_LFS_9901. Accessed: 2025-01-17, 16:16:00 (2025).

[62] Eurostat. Tps_00001 dataset from Eurostat database, 10.2908/TPS00001. Accessed: 2025-01-17, 16:44:00 (2025).

[63] Federal Statistical Office (Destatis). Data retrieved from GENESIS online database, https://www-genesis.destatis.de. Accessed: 2025-01-20, 09:59:36 (2024).

[64] R Core Team. *R: A Language and Environment for Statistical Computing*. Vienna, Austria (2024).

[65] Wickham, H. *et al*. Welcome to the Tidyverse. *Journal of Open Source Software* **4**, 1686 10.21105/joss.01686 (2019).

[66] Lüdecke, D.* sjlabelled: Labelled Data Utility Functions (Version 1.2.0)*, 10.5281/zenodo.1249215 (2022).

[67] Lüdecke, D. *sjmisc: Data and Variable Transformation Functions*. R package version 2.8.10 (2024).

[68] Lumley, T.* survey: Analysis of Complex Survey Samples*. R package version 4. 4-2 (2024).

[69] Cannell, B.* codebookr: Create Codebooks from Data Frames*. R package version 0.1.9 (2026).

[70] Yentes, R. & Wilhelm, F.* careless: Procedures for computing indices of careless responding*. R package version 1.2.2 (2023).

[71] Tierney, N. & Cook, D. Expanding tidy data principles to facilitate missing data exploration, visualization and assessment of imputations. *Journal of Statistical Software* **105**, 1–31 10.18637/jss.v105.i07 (2023).

[72] Helbling, M., Fadarkhan Osenbrügge, N. & Jungkunz, S. Radical and Extremist Attitudes in Europe, 10.4232/1.14814 (2026).42892640

[73] Greszki, R., Meyer, M. & Schoen, H. Exploring the Effects of Removing “Too Fast” Responses and Respondents from Web Surveys. *Public Opinion Quarterly* **79**, 471–503 10.1093/poq/nfu058 (2015).

[74] Curran, P. G. Methods for the detection of carelessly invalid responses in survey data. *Journal of Experimental Social Psychology* **66**, 4–19 10.1016/j.jesp.2015.07.006 (2016).

[75] Chen, F. F. What happens if we compare chopsticks with forks? The impact of making inappropriate comparisons in cross-cultural research. *Journal of Personality and Social Psychology* **95**, 1005–1018 10.1037/a0013193 (2008). 18954190

[76] Greenwald, A. G. *et al*. Best research practices for using the Implicit Association Test. *Behavior Research Methods* **54**, 1161–1180 10.3758/s13428-021-01624-3 (2022). PMC9170636 34519017

[77] Lane, K. A., Banaji, M. R., Nosek, B. A. & Greenwald, A. G. Understanding and Using the Implicit Association Test: IV: What We Know (So Far) about the Method. In Wittenbrink, B. & Schwarz, N. (eds.) *Implicit Measures of Attitudes*, 59–102 (Guilford Press, New York, 2007).

[78] Greenwald, A. G. & Lai, C. K. Implicit Social Cognition. *Annual Review of Psychology* **71**, 419–445 10.1146/annurev-psych-010419-050837 (2020). 31640465

[79] Gawronski, B., Galdi, S. & Arcuri, L. What Can Political Psychology Learn from Implicit Measures? Empirical Evidence and New Directions: Implicit Measures in Political Psychology. *Political Psychology* **36**, 1–17 10.1111/pops.12094 (2015).

[80] Hilgenstock, M., Jedinger, A., Kolkwitz-Anstötz, P. & Siegers, P. DP-R∣EX: Das Datenportal für die Rassismus- und Rechtsextremismusforschung. *ZRex - Zeitschrift für Rechtsextremismusforschung* **5**, 312–316 10.3224/zrex.v5i2.09 (2025).

